# Relationships between diet and gut microbiome in an Italian and Dutch cohort: does the dietary protein to fiber ratio play a role?

**DOI:** 10.1007/s00394-023-03308-4

**Published:** 2023-12-27

**Authors:** Silvia Tagliamonte, Marie-Luise Puhlmann, Francesca De Filippis, Mathilde Guerville, Danilo Ercolini, Paola Vitaglione

**Affiliations:** 1 Department of Agricultural Sciences, University of Naples Federico II, Parco Gussone Ed. 84, 80055 Portici, Italy; 2 Task Force On Microbiome Studies, University of Naples Federico II, 80134 Naples, Italy; 3 Division of Human Nutrition and Health, Wageningen University and Research, Wageningen, The Netherlands; 4 Laboratory of Microbiology, Wageningen University and Research, Wageningen, The Netherlands; 5 Nutrition Department, Lactalis Research and Development, 35240 Retiers, France

**Keywords:** Dietary fiber, Proteins, Nutrient ratios, Gut microbiome, Gene richness

## Abstract

**Purpose:**

To investigate the relationships between the habitual diet, the protein to fiber ratio (P/F), and the gut microbiome in one Italian and one Dutch cohort of healthy subjects consuming an omnivore diet.

**Methods:**

The Italian cohort included 19 males (M_IT, BMI 25.2 ± 0.72 kg/m^2^, age 25.4 ± 0.96 years) and 20 females (F_IT, BMI 23.9 ± 0.81 kg/m^2^, age 23.8 ± 0.54 years); the Dutch cohort included 30 females (F_NL, BMI: 23.9 ± 0.81 kg/m^2^, age: 23.8 ± 0.54 years). Individual diets were recorded through Food Frequency Questionnaires and analyzed to assess the nutrient composition. Gut microbiome was assessed in fecal samples.

**Results:**

M_IT consumed higher levels of proteins than F_NL and F_IT, whereas dietary fiber intake did not differ among groups. Data showed that consumption of plant protein to animal protein (PP/AP) and PP to total proteins ratio can determine a differentiation of F_NL more than the absolute amount of dietary fiber. Conversely, the protein to fiber (P/F) and AP to total proteins better characterized M_IT. M_IT harbored the highest abundance of proteolytic microorganisms and the lowest microbial gene richness. Conversely, F_NL had more fiber-degrading microorganisms like *Bacteroides thetaiotaomicron*, *Bacteroides xylanisolvens, Roseburia* sp., *Coprococcus eutactus* and *Parabacteroides* along with the highest number of genes encoding carbohydrate-active enzymes and gene richness. It was predicted that by each unit decrease in the P/F a 3% increase in gene richness occurred.

**Conclusion:**

Study findings suggested that dietary P/F, rather than the absolute amount of dietary fiber, could contribute to the shaping of the microbiome towards a more proteolytic or fiber-degrading gut ecosystem.

*ClinicalTrials.gov Identifier* NCT04205045—01-10-2018, retrospectively registered.

*Dutch Trial Register* NTR7531—05-10-2018.

**Supplementary Information:**

The online version contains supplementary material available at 10.1007/s00394-023-03308-4.

## Introduction

The incidence of chronic diseases such as diabetes and cardiovascular disease is dramatically increasing worldwide, having reached epidemic proportions [1]. Diet is central to human health and gut microbiome is a key player in host’s metabolic balance, as gut microbiota dysbiosis is considered as an extra-genetic risk factor of many chronic diseases [2, 3]. Gut microbiome plays a role in food digestion and nutrient absorption by breaking down complex molecules reaching the large intestine, orchestrating the mucosal immune response and synthesizing a plethora of bioactive compounds [4]. Diet constitutes a major factor shaping human gut microbiome [5].

Results from randomized controlled trials frequently show that the health effects of a nutritional intervention may not be consistently reproducible at the population level. This inconsistency is often attributed to the high variability in individual responses to similar diets. This phenomenon may rely on the actual status of body systems including the gut microbiome, the cross-talk ability of the involved systems and their responsiveness to the dietary factors [6, 7]. Evidence also shows sex differences occurring between men and women in the gut microbiome because of testosterone and estrogens levels [8] and/or the habitual diet [9]. Sex-specific differences in response to dietary fiber consumption have been reported [9, 10].

Clarifying the factors of the diet and microbiome affecting the physiological response in humans is pivotal to define effective personalized nutrition and precision medicine strategies that positively influences health and reduces the disease risk [4, 11].

Numerous studies have investigated the relationship between dietary fiber intake and various health outcomes, showing conflicting conclusions, as mostly fails to consider how dietary fibers are consumed daily and their effects on the digestive tract as intrinsic structures within plant tissues [12]. The effects of a single nutrient on gut microbiome may be enhanced or counteracted by other nutrients [13]. There is consistent evidence to support the notion that lowering dietary protein to fiber intake may affect the gut microbiome thus lowering the risk of cardiovascular disease (CVD) [14, 15]. An estimated 12–18 g of dietary proteins reach the large intestine daily, where they may undergo microbial fermentation. This fermentation process can yield metabolites that are associated with various gastrointestinal diseases [16, 17]. Reducing protein intake while increasing dietary fiber intake through fruit, vegetables and wholegrain consumption may determine a shift from a proteolytic to saccharolytic intestinal fermentation. As a result, there may be a decrease in protein-derived uremic toxins generation [18–20]. Mounting evidence showed that uremic toxins, i.e., indoxyl sulfate and p-cresyl sulfate may contribute to CVD and bone disease [21]. It was demonstrated that lower is the protein to fiber ratio intake the lower is the generation of uremic toxins and systemic inflammation in patients suffering from chronic kidney disease [15, 22]. Recently, it was shown that healthy subjects and patients with insulin-resistance habitually consuming more protein at the expense of fiber were more responsive to increase the gut microbiome capability to ferment fiber than subjects with lower protein to fiber ratio; this resulted in a decrease of blood cholesterol levels [23].

Despite the association between uremic toxins production and dietary protein to fiber intake in both healthy and non-healthy subjects being well established, its relationship with microbiome signature as well as, gut microbial gene richness has been underexplored. We hypothesized that the protein to fiber ratio in the habitual diet could affect the gut microbiome composition and functionality.

This study aimed at exploring the impact of the diet and the protein to fiber ratio in driving differences in gut microbiome in healthy people from two different cohorts habitually consuming an omnivore diet. To enlarge and diversify the analysis we compared the dietary and gut microbiota traits of an Italian cohort with a Dutch cohort.

## Materials and methods

### Study design and subject characterization

This study involves two cohorts of subjects, one from Italy and one from The Netherlands, who participated in two studies that were conducted at the same time and shared the same criteria (except sex) for the selection of subjects aiming at exploring the physiological mechanisms underpinning cow’s milk digestion by focusing on post-prandial gastro-intestinal responses or gastric emptying, respectively [24, 25]. In the present study, the combination of data on diets and gut microbiome collected over the two main studies was performed to increase the sample size and statistical power. This approach aimed to bolster the robustness of the analysis and reliability of the findings using a dataset possibly providing a broader variability in the investigated dietary factors.

The study was conducted after the approval of the University of Naples Ethic Committee (Protocol number: 177/18) and the Medical Ethical Review Committee Wageningen University (METC) (number: 18/17) for Italian and Dutch cohort, respectively. Each participant signed a written informed consent. The trials were registered at www.clinicaltrials.gov (number NCT04205045) and Dutch Trial Register (number NTR7531) for Italian and Dutch cohort, respectively. The two cohorts included healthy, adult subjects, without food allergy/intolerance, and having an omnivorous diet [24, 25]. The Italian cohort included males and females whereas the Dutch cohort was of only females. Anthropometric characterization of the participants consisted of body weight and height measurements. Individual diets were recorded through an 110-item Food Frequency Questionnaire (FFQ) for the Italian cohort [26] and an 183-item FFQ for Dutch cohort [27, 28]. The databases used to estimate the nutrients intake were the BDA (Banca Dati di composizione degli Alimenti) or CREA (Consiglio per la Ricerca in agricoltura e l'analisi dell'Economia Agraria) Italian food databases, the U.S. Department of Agriculture (USDA) National Nutrient Database and the Dutch food composition database Nederlands Voedingsstoffenbestand (NEVO). Fasting participants provided a fecal sample collected according to the standard operating procedure (SOP 004) of the International Human Microbiome Standards (IHMS) (www.microbiome-standards.org) for the gut microbiome analysis.

### Gut microbiome

The gut microbiome was analyzed by shotgun metagenomics as previously reported [24]. DNA libraries were sequenced on an Illumina NovaSeq platform (Illumina, San Diego, California, USA), leading to 2 × 150 bp, paired-end reads. Human reads were removed using the Human Sequence Removal pipeline developed within the Human Microbiome Project using the Best Match Tagger (BMtagger; https://hmpdacc.org/hmp/doc/HumanSequenceRemoval_SOP.pdf). The resulting reads were quality-checked and filtered through Prinseq-lite v0.20.4 (with -trim_qual_right 5 and -min_len 60 parameters) [29]. Taxonomic and metabolic profiles were estimated using MetaPhlAn v3.0 and HUMAnN v3.0, respectively [30]. The *diversity* function (from the R package *‘vegan’*) was applied on species-level taxonomic profiles to estimate Shannon–Wiener and Simpson’s α-diversity indices. Metagenomics reads were assembled, and genes were predicted from contigs > 1000 bp using a pipeline recently described [24]. Predicted genes were aligned against the carbohydrate-active enzymes database CAZY (URL http://www.cazy.org/) using DIAMOND v2.0.4 (–very_sensitive option [31]), with options and cutoff threshold previously described [24]. Gene abundance was obtained using the Reads Per Kilobase per Million of Reads (RPKM) method [32]. Microbial gene richness was calculated as described by Le Chatelier et al. (2013) [33].

### Statistical analysis

Statistical analysis and visualization were carried out in R version 4.0.3 (https://www.r-project.org). After variables were checked for normality, significantly skewed variables were natural-log transformed [ln(x + k), with k values zeroing the skewness]. For variables with a normal distribution according to the Shapiro–Wilk test, a One-way ANOVA with Tukey’s post hoc was performed to assess differences between groups. For non-parametric variables, the Mann–Whitney test was conducted to detect between-group differences. Two-tailed P-values lower than 0.05 were considered significantly different. Data are expressed as the means ± standard errors (SEMs). A principal component analysis (PCA, *pca* function) was performed to explore differences in habitual diet macronutrient composition (library FactoMineR). Moreover, statistical significance of the distance between group centroids in PCA analysis was computed using the Hotelling T2 test (library Hotelling).

To explore differences in gut microbiome profiles, a linear discriminant analysis (LDA) effect size (LEfSE) was applied [34]. In addition, the regression plot was visualized using scatter plots (ggscatter function, ggplot2 R package) [35]. Linear regression was performed using *lm* and *predict* function (stats package) [36] to predict the effect of one or more predictor variables on a microbial gene richness outcome. The model was built by splitting data in training data (80%) and testing (20%). The model K-fold cross-validation was performed with k = 10 (caret package) [37].

## Results

### Subject characteristics and habitual diet

Table 1 shows the general characteristics and the habitual diet of the subjects grouped by nationality and sex with 19 Italian males (average BMI: 25.2 ± 0.72 kg/m^2^, age: 25.4 ± 0.96 years), 20 Italian females (average BMI: 23.9 ± 0.81 kg/m^2^, age: 23.8 ± 0.54 years) and 30 Dutch females (average BMI: 23.9 ± 0.81 kg/m^2^, age: 23.8 ± 0.54 years).

**Table 1:** General characteristics of Italian female cohort (F_IT), Dutch female cohort (F_NL), Italian male cohort (M_IT)

	Italian females (*n* = 20)	Dutch females (*n* = 30)	Italian males (*n* = 19)
Age	23.8 ± 0.54^a^	25.2 ± 0.95^a^	25.4 ± 0.96^a^
Body weight (kg)	62.1 ± 2.04^b^	66.5 ± 1.56^b^	77.5 ± 2.96^a^
BMI (kg/m^2^)	23.9 ± 0.81^ab^	22.4 ± 0.42^b^	25.2 ± 0.72^a^
Habitual diet			
Energy intake (kcal/day)	1698.9 ± 136.16^b^	1939.6 ± 109.01^a,b^	2399.8 ± 161.73^a^
Proteins (g/day)	79.7 ± 6.45^b^	68.4 ± 4.46^b^	105.4 ± 7.22^a^
Fats (g/day)	68.4 ± 5.86^b^	82.7 ± 5.30^a,b^	98.2 ± 7.64^a^
Carbohydrates (g/day)	174.2 ± 16.07^b^	209.4 ± 11.78^a,b^	255.6 ± 19.59^a^
Dietary Fiber (g/day)	25.6 ± 3.07^a^	24.4 ± 1.46^a^	21.9 ± 1.60^a^
Protein/Fiber	3.4 ± 0.2^b^	2.9 ± 0.12^b^	5.1 ± 0.39^a^

Different letters indicate *p* < 0.05 assessed by One-way ANOVA and Tukey’s post hoc test. Data are expressed as means ± SEM

The three groups were homogeneous for age, but males showed a slightly higher BMI than Dutch females. With regards to habitual diet, Italian males consumed significantly higher amounts of energy, carbohydrates and fats than the Italian females, whereas the macronutrient intake of Dutch females did not differ from either of the two groups. M_IT consumed more proteins compared to F_IT and F_NL, while the absolute amount of dietary fibers was homogeneous among groups. The protein to fiber ratio was higher in M_IT followed by F_IT and F_NL who showed a similar ratio (Table 1). However, when the dietary energy coming from the different macronutrients was considered, data showed that the contribution of carbohydrates and fats was similar between the groups (Supplementary Table 1). Conversely the Italians (both males and females) consumed significantly higher energy from proteins than F_NL and the energy from dietary fiber was ranked as F_IT > F_NL > M_IT.

**Fig. 1:**
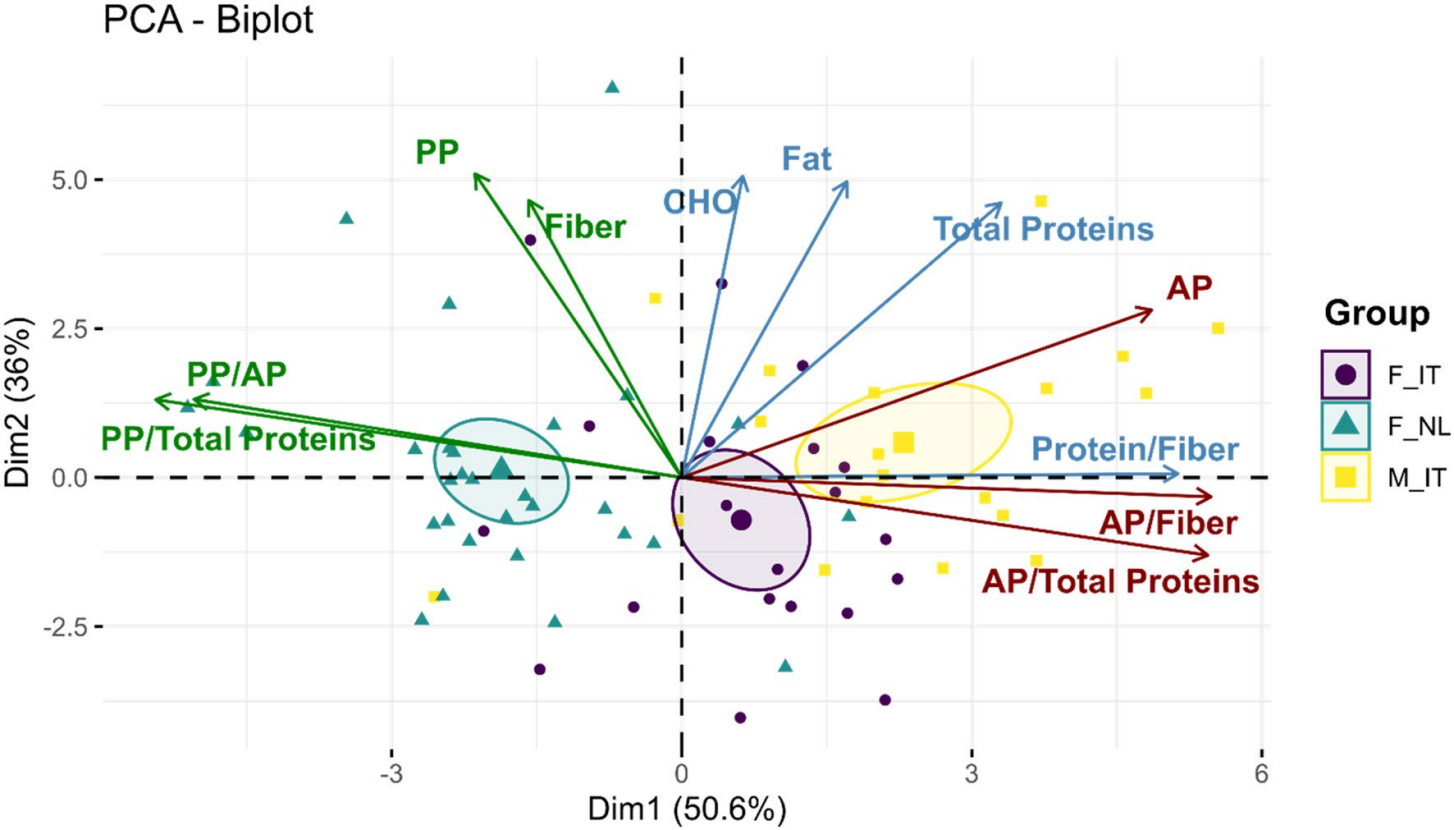
Principal component analysis biplot based on habitual macronutrients intake and Protein/Fiber ratio in Italian female cohort (F_IT; light violet), Dutch female cohort (F_NL; teal), Italian male cohort (M_IT; yellow). CHO, Carbohydrates; PP, Plant proteins; AP, Animal proteins. Vectors representing plant and animal products are shown in green and red, respectively. Between group distances were assessed by computing Mahalanobis distance and Hotelling’s T2 test

The intake of plant proteins (PP) and animal proteins (AP) along with the total proteins and in relation to the dietary fiber intake were considered in computing a principal component analysis (Fig. 1). Data showed that PP/AP ratio, PP/total proteins ratio, protein/fiber ratio and AP/fiber ratio contributed to the separation of the three cohorts more than the absolute intake of dietary fibers and proteins. Protein to fiber ratio and AP to fiber ratio better characterized M_IT participants with F_IT lying midway between M_IT and F_NL. These observations were confirmed by the statistical significance of the distance between groups computed by the Hotelling T2 test showing that M_IT *vs* F_NL centroids distance of T2 = 35 (*p*-value ≤ 0.001).

Furthermore, the lowest protein to fiber ratio shown by F_NL resulted from a significantly higher intake of whole grain products and a significantly lower intake of fish products compared to M_IT and F_IT (Supplementary Fig. 1). Participants from M_IT group consumed more of refined grain products, milk and dairy products as well as processed meat than F_IT and F_NL (Supplementary Fig. 1).

### Individual dietary protein-to-fiber ratio differentiates the gut microbiome with Dutch females harboring more fiber-degrading and Italian males more proteolytic microorganisms

**Fig. 2:**
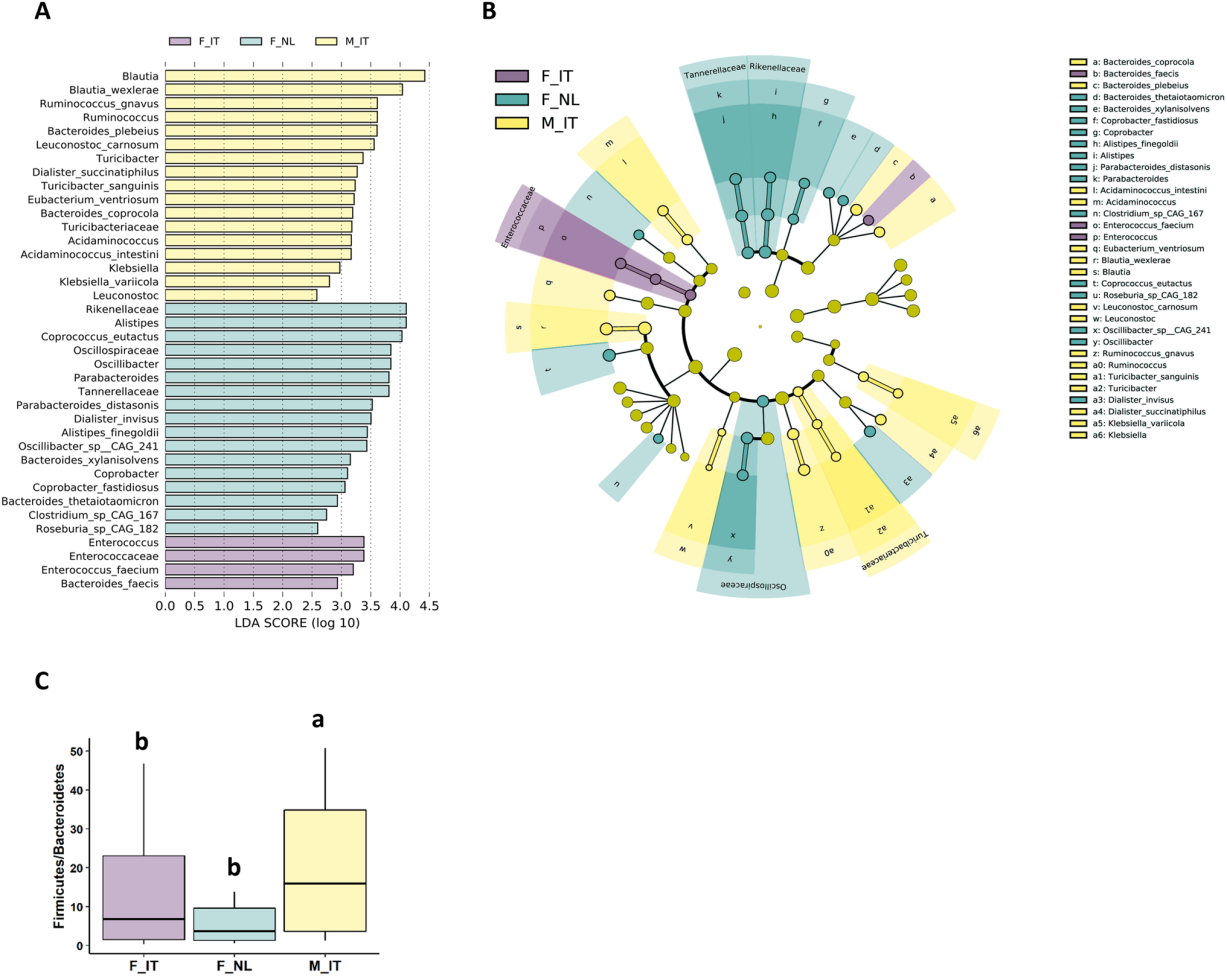
Linear discriminant analysis effect size (LEfSe) (**A**) and Cladogram visualization (**B**) showing the differentially abundant species, genus and families between in Italian female cohort (F_IT; light violet), Dutch female cohort (F_NL; teal), Italian male cohort (M_IT; yellow). The bacterial taxa shown exhibited a statistically significant change (*p* < 0.05) when the logarithmic linear discriminant analysis (LDA) score threshold was set to 2. (**C**) Firmicutes/Bacteroidetes ratio in Italian female cohort (F_IT; light violet), Dutch female cohort (F_NL; teal), Italian male cohort (M_IT; yellow). The box plots show the data distribution based on the first quartile, median and third quartile. Different letters indicate *p* < 0.05 assessed by Wilcoxon test

The Linear discriminant analysis (LDA) Effect Size (LEfSe) analysis and cladogram plot were generated to assess the effect size of each differentially abundant taxon in the gut among groups (Fig. 2a, b). M_IT showed higher abundance of *Bacteroides* spp., *Blautia, Klebsiella* and *Ruminococcus gnavus,* along with higher Firmicutes/Bacteroidetes ratio (Fig. 2c). Seventeen taxa were significantly enriched in F_NL such as *Bacteroides thetaiotaomicron*, *Bacteroides xylanisolvens*, *Roseburia* sp. CAG 182, *Coprococcus eutactus* and *Parabacteroides*. Only four taxa were significantly enriched in F_IT mostly belonging to the *Enterococcaceae* family.

To explore the saccharolytic pattern of the gut microbiome, all-against-all LEfSe analysis on genes coding for carbohydrate-active enzymes (CAZymes) was performed (Table 2). Results showed a higher number of CAZymes in F_NL (*n* = 16) followed by F_IT (*n* = 8) and M_IT (*n* = 4). Specifically, 10 out of 16 CAZymes significantly enriched in Dutch females *vs* F_IT and M_IT were glycoside hydrolase, polysaccharide lyase and carbohydrate esterase while 50% of CAZymes enriched in F_IT were carbohydrate-binding modules (CBMs).

**Table 2:** Linear discriminant analysis effect size (LEfSe) all-against-all showing the differentially abundant genes encoding carbohydrate active enzymes (CAZymes)

Enzyme families	Activity	Enriched group	Lefse	*p*-value
CBM32	Binding to polygalaturonic acid components of pectin, to galactose and lactose, LacNAc (β-D-galactosyl-1,4-β-D-N-acetylglucosamine) shown for certain bacteria	F_IT	2.71	0.043
CBM0	Unclassified cmb	F_IT	2.25	0.002
GH84	N-acetyl β-glucosaminidase (EC 3.2.1.52); hyaluronidase (EC 3.2.1.35); [protein]-3-O-(GlcNAc)-L-Ser/Thr β-N-acetylglucosaminidase (EC 3.2.1.169)	F_IT	2.23	0.026
GH101	endo-α-N-acetylgalactosaminidase (EC 3.2.1.97)	F_IT	2.20	0.023
GH85	endo-β-N-acetylglucosaminidase (EC 3.2.1.96)	F_IT	2.08	0.017
CBM51	Attached to various enzymes from families GH2, GH27, GH31, GH95, GH98 and GH101	F_IT	2.05	0.002
AA10 (FORMERLY CBM 33)	Copper-dependent lytic polysaccharide monooxygenases (LPMOs); some proteins have been shown to act on chitin, others on cellulose	F_IT	1.78	0.006
CBM68	Binding to maltotriose and maltotetraose, binding to galactose for certain bacteria	F_IT	1.33	0.050
GH20	β-hexosaminidase (EC 3.2.1.52); lacto-N-biosidase (EC 3.2.1.140); β-1,6-N-acetylglucosaminidase (EC 3.2.1.-); β-6-SO3-N-acetylglucosaminidase (EC 3.2.1.-)	F_NL	3.19	0.013
GH89	α-N-acetylglucosaminidase (EC 3.2.1.50)	F_NL	2.57	0.003
GH110	α-galactosidase (EC 3.2.1.22); α-1,3-galactosidase (EC 3.2.1.-)	F_NL	2.40	0.018
GH123	β-N-acetylgalactosaminidase (EC 3.2.1.53); glycosphingolipid β-N-acetylgalactosaminidase (EC 3.2.1.-)	F_NL	2.37	0.001
PL38	endo-β-1,4-glucuronan lyase (EC 4.2.2.14)	F_NL	2.15	0.004
PL35	chondroitin lyase / chondroitinase (EC 4.2.2.-)	F_NL	2.02	0.005
PL26	rhamnogalacturonan exolyase (EC 4.2.2.24)	F_NL	2.00	0.035
CBM67	L-rhamnose binding activity shown for certain bacteria	F_NL	2.00	0.002
GT101	β-glucosyltransferase (EC 2.4.1.-)	F_NL	1.95	< 0.001
GH50	β-agarase (EC 3.2.1.81)	F_NL	1.95	0.011
PL15	alginate lyase (EC 4.2.2.3); oligoalginate lyase / exo-alginate lyase (EC 4.2.2.26); heparin lyase / heparin lyase I (EC 4.2.2.7); heparin-sulfate lyase / heparin lyase III (EC 4.2.2.8)	F_NL	1.90	0.005
PL29	chondroitin-sulfate ABC endolyase (EC 4.2.2.20)	F_NL	1.85	0.009
GT80	β-galactoside α-2,6-sialyltransferase (EC 2.4.99.1); β-galactoside α-2,3-sialyltransferase (EC 2.4.99.4)	F_NL	1.54	0.001
CE3	acetyl xylan esterase (EC 3.1.1.72)	F_NL	1.44	0.009
GH99	glycoprotein endo-α-1,2-mannosidase (EC 3.2.1.130); mannan endo-1,2-α-mannanase (3.2.1.198)	F_NL	1.31	0.001
GT21	UDP-Glc: ceramide β-glucosyltransferase (EC 2.4.1.80)	F_NL	1.13	0.049
GH77	amylomaltase or 4-α-glucanotransferase (EC 2.4.1.25)	M_IT	3.02	0.036
GH25	lysozyme (EC 3.2.1.17)	M_IT	2.88	0.023
GH120	β-xylosidase (EC 3.2.1.37)	M_IT	2.57	0.012
CBM22	A xylan binding function has been demonstrated in several cases and affinity with mixed β-1,3/β-1,4-glucans in one	M_IT	2.43	0.006

GH, Glycoside Hydrolases; GT, Glycosyl Transferases; PL, Polysaccharide Lyases; CE, Carbohydrate Esterases; AA, Auxiliary Activities; CBM, Carbohydrate-Binding Modules

### The dietary protein/fiber ratio predicts the gut microbiome gene richness

**Fig. 3:**
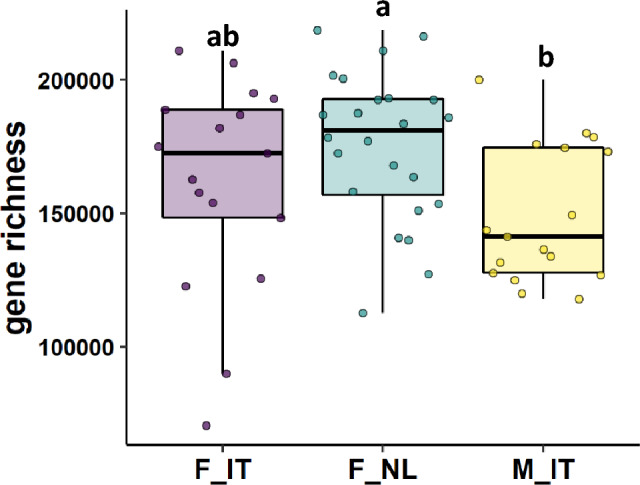
Gut microbial gene richness in Italian female cohort (F_IT; light violet), Dutch female cohort (F_NL; teal), Italian male cohort (M_IT; yellow). The box plots show the data distribution based on the first quartile, median and third quartile. Different letters on the boxplot indicate significant between-group differences (*p*-value < 0.05) assessed by One-way ANOVA and Tukey’s post hoc test

The gut microbial gene richness in the three groups is shown in Fig. 3. F_NL showed the highest gene richness, while F_IT only showed a trend toward higher gene richness compared to M_IT. The α-diversity was not found to be significantly different among groups (Supplementary Fig. 2). However, F_NL showed a trend toward higher α-diversity (Shannon index) compared to M_IT.

**Fig. 4:**
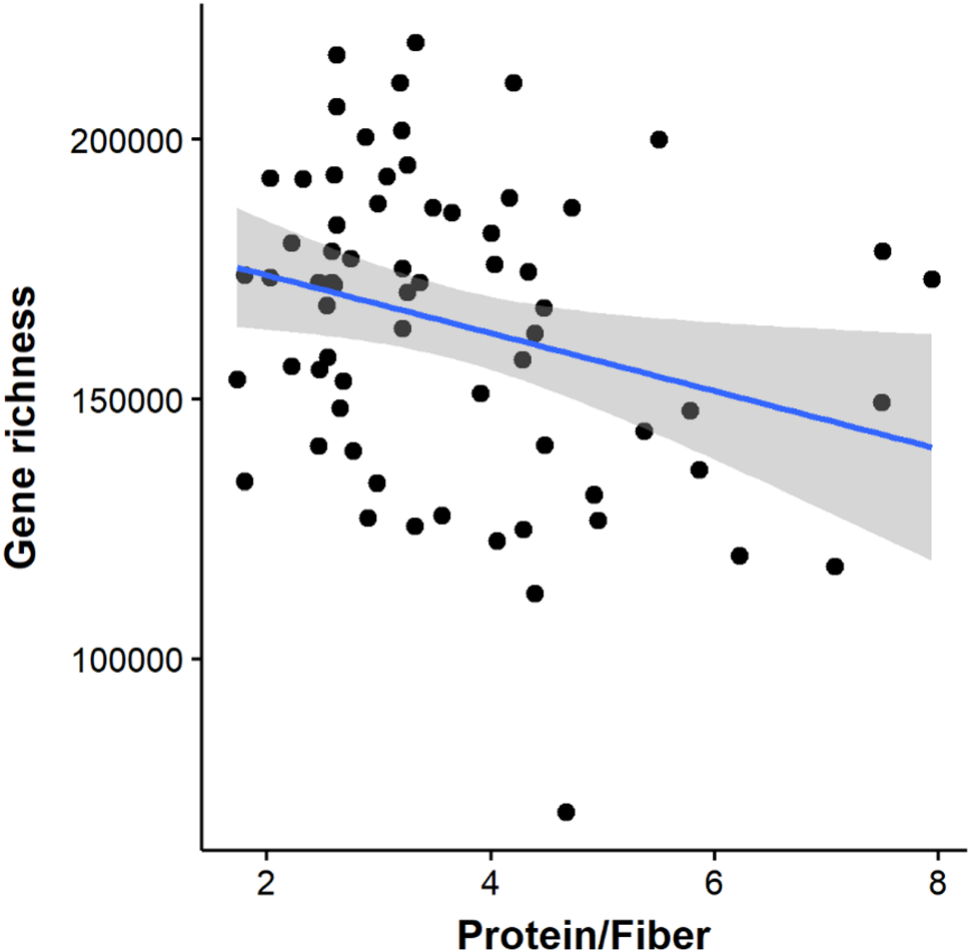
Linear regression analysis to test whether the protein/fiber predicted the gut microbial gene richness after controlling for sex. The model min–max accuracy was 0.86; mean absolute percentage deviation (MAPE) = 0.16. Upon k-fold cross-validation with k = 10, R-squared = 0.25, Mean absolute error (MAE) = 26,543.19,* p*-value = 0.04

Figure 4 shows a linear regression model of the gene richness as the dependent variable on protein/fiber ration intake. The result of the regression indicated that the predictor explained 12% of variance (Adjusted R-squared = 0.092, *p*-value = 0.016) while prediction accuracy was 86%. No association was found between energy intake from fiber (r = 0.21, *p* = 0.11) or absolute amount of fiber (r = 0.13, *p* = 0.32) or other macronutrients (*data not shown*) and gene richness.

## Discussion

The findings of this study showed that the dietary protein to fiber ratio had a distinct impact on the gut microbiome signature that was not attributable to the dietary fiber alone. Females (NL, IT) and males (IT) consumed diets that differed in the protein to fiber ratio and in the contribution of animal or plant proteins to total proteins but were similar in overall dietary fiber. Notwithstanding, females and males exhibited differences in the gut microbiota composition and potential capability to ferment dietary fiber.

Participants from M_IT group consumed a diet with the highest protein to fiber ratio and animal proteins and, in comparison to the other groups, they harbored in the gut a higher level of *Bacteroides* spp., *Blautia, Klebsiella* genus and *Ruminococcus gnavus* previously associated with animal protein intake, saturated fat and low consumption of plant proteins [38–40]. Specifically, L-*Ruminococcus*, has been found to mirror fat intake, and urinary excretion of TMAO associated to the consumption of eggs, beef, pork and fish [41]. Despite functional differences between species of *Bacteroides*, they are generally recognized as primary protein hydrolyzers, making amino acids and peptides available for both facultative saccharolytic bacteria and obligate amino acid fermenters [42]. The highest abundance of *Klebsiella*, Family XIII Clostridiales, some members of Lachnospiraceae as well as the opportunistic pathogen *Acidaminococcus* found in M_IT, suggests a more proteolytic gut microbiome [42, 43] in line with the highest dietary protein to fiber ratio. Evidence shows that a proteolytic gut microbiome is detrimental for health due to both the production of toxic metabolites and flourishing environment for the growth of opportunistic pathogens and pro-inflammatory microorganisms [42, 44]. For instance, in patients suffering from chronic kidney disease the higher is the dietary protein to fiber ratio the higher is the generation of gut microbiome-derived uremic toxins and systemic inflammation [15, 22]. *R. gnavus* is known for the mucolytic activity (utilizing glycans from the intestinal mucus layer as energy sources) damaging the gut barrier integrity [45].

The hypothesis of a more proteolytic gut microbial ecosystem in M_IT than F_IT and F_NL was further strengthened by the finding of a higher Firmicutes/Bacteroidetes ratio. Firmicutes has been associated with a high intake of animal products [40] whereas Bacteroidetes is known for harvesting energy from starch and fiber breakdown [46, 47].

In agreement with the lowest dietary protein/fiber ratio, F_NL showed in the gut the highest proportion of short-chain fatty acid-producing and fiber-degrading microorganisms such as *Bacteroides thetaiotaomicron*, *Bacteroides xylanisolvens*, *Roseburia* sp. CAG 182, *Coprococcus eutactus* and *Parabacteroides* [23, 38, 40, 41, 48]. It was recently shown that the fiber degrading *B. thetaiotaomicron,* inhibits indole production thus limiting the generation of the uremic toxins [20]. Participants from F_NL group who consumed the highest amount of snacks, also showed the highest levels of *Alistipes* genus which was associated with fast food or ready meal consumption [38]. Although *Alistipes* is known to be proteolytic fermenter, no correlation was found with protein intake in omnivores and vegans [49]. The high intestinal level of *Oscillobacter* genus in F_NL might result from the high intake of tea phenols and plant proteins which are believed to increase *Oscillobacter* genus at the expense of pro-inflammatory microorganisms [38, 49]. F_IT only showed a gut microbiome more enriched in the probiotics Enterococcaceae family, possibly associated to dairy products intake [50].

Dutch females also harbored in the gut a higher proportion of genes coding for carbohydrate-active enzymes (CAZymes) being 9% of all CAZymes more abundant in F_NL, 4% in F_IT and, only 2% in M_IT. In particular, most CAZymes enriched in F_NL were related to fiber breakdown (i.e., glycoside hydrolase, polysaccharide lyase and carbohydrate esterase), strengthening the hypothesis of a more saccharolytic gut microbiome. Moreover, 10 out of 16 CAZymes in F_NL were putatively involved in the breakdown of plant cell wall fiber, starch, inulin and pectins [51–55]. According to gut microbiome composition, those CAZymes were primarily attributable to *B. thetaiotaomicron* and *Alistipes*. Participants from M_IT group showed the lowest proportion of genes encoding CAZymes while F_IT mostly showed higher proportion of carbohydrate-binding modules (CBMs) which did not possess carbohydrates catalytic activity of their own [55]. This result supported the hypothesis that the habitual consumption of a diet with a high protein to fiber ratio can lead to a decreased saccharolytic capability of the gut microbiome.

Participants from F_NL group showed the highest gene richness while the α-diversity did not differ among groups. It is believed that α-diversity tends to be greater in females than males partly depending on sex-hormones differences [9]. Nevertheless, discrepancies are present in the literature with some studies reporting no sex differences in α-diversity [9, 56, 57]. Despite sex-hormones may have a role in shaping gut microbiome, diet is one of most impactful modulators of the gut microbiota composition [9].

Findings of this study also showed that dietary protein to fiber ratio, rather than dietary fiber alone, predicts the gut microbial gene richness; each unit decrease in the protein to fiber ratio corresponded to 3% increase in gene richness.

This study has two limitations. Firstly, the involvement of a small number of participants. Secondly, the absence of a group of males in the Dutch cohort may be seen as a limitation as we could not exclude an influence of the sex in the gut microbial and gene richness differences found between M_IT and F_NL. However, sex-diet interactions were out of scopes of this study.

In conclusion, the results of this study involving two cohorts from two different countries suggested that dietary protein to fiber ratio, rather than dietary fiber alone, significantly influences the composition of the microbiome, potentially shifting it towards a more proteolytic or fiber-degrading gut ecosystem as well as a different gene richness.

Intervention studies designed to reduce the dietary protein to fiber ratio or increase the dietary fiber intake are needed to demonstrate whether and at which extent the two approaches may affect gut microbiome composition and health outcomes. Such a confirmation would reveal opportunities for clinical practice in the field of personalized nutrition, for instance applying effective changes in dietary protein to fiber ratio may modulate gut microbiome composition and improve individual health and wellbeing according to the needs.

### Supplementary Information

Below is the link to the electronic supplementary material.

Supplementary file1 (DOCX 590 KB)

## Data Availability

The raw sequence reads generated in this study have been deposited in the Sequence Read Archive (SRA) of the NCBI under accession number PRJNA832737.

## References

[1] Jaacks LM, Vandevijvere S, Pan A, McGowan CJ, Wallace C, Imamura F, Mozaffarian D, Swinburn B, Ezzati M (2019). The obesity transition: stages of the global epidemic. Lancet Diabetes Endocrinol.

[2] Cox LM, Blaser MJ (2013). Pathways in microbe-induced obesity. Cell Metab.

[3] Claus SP (2013). Fighting undernutrition: don’t forget the bugs. Cell Host Microbe.

[4] Kolodziejczyk AA, Zheng D, Elinav E (2019). Diet-Microbiota interactions and personalized nutrition. Nat Rev Microbiol.

[5] Cuevas-Sierra A, Ramos-Lopez O, Riezu-Boj JI, Milagro FI, Martinez JA (2019). Diet, gut microbiota, and obesity: Links with host genetics and epigenetics and potential applications. Adv Nutr.

[6] van Ommen B, van den Broek T, de Hoogh I, van Erk M, van Someren E, Rouhani-Rankouhi T, Anthony JC, Hogenelst K, Pasman W, Boorsma A, Wopereis S (2017). Systems biology of personalized nutrition. Nutr Rev.

[7] Zeevi D, Korem T, Zmora N, Israeli D, Rothschild D, Weinberger A, Ben-Yacov O, Lador D, Avnit-Sagi T, Lotan-Pompan M, Suez J, Mahdi JA, Matot E, Malka G, Kosower N, Rein M, Zilberman-Schapira G, Dohnalová L, Pevsner-Fischer M, Bikovsky R, Halpern Z, Elinav E, Segal E (2015). Personalized nutrition by prediction of glycemic responses. Cell.

[8] Yoon K, Kim N (2021). Roles of sex hormones and gender in the gut microbiota. J Neurogastroenterol Motility.

[9] Kim N (2022) Sex difference of gut microbiota. Sex/Gender-Specific Medicine in the Gastrointestinal Diseases 363–377. 10.1007/978-981-19-0120-1_22

[10] Dominianni C, Sinha R, Goedert JJ, Pei Z, Yang L, Hayes RB, Ahn J (2015). Sex, body mass index, and dietary fiber intake influence the human gut microbiome 10:e0124599. PLoS ONE.

[11] De Filippis F, Vitaglione P, Cuomo R, Berni Canani R, Ercolini D (2018). Dietary interventions to modulate the gut microbiome—how far away are we from Precision Medicine. Inflamm Bowel Dis.

[12] Puhlmann M-L, de Vos WM (2022). Intrinsic dietary fibers and the gut microbiome: rediscovering the benefits of the plant cell matrix for human health. Front Immunol.

[13] Zmora N, Suez J, Elinav E (2018). You are what you eat: Diet, health and the gut microbiota. Nat Rev Gastroenterol Hepatol.

[14] Packard DP, Milton JE, Shuler LA, Short RA, Tuttle KR (2006). Implications of chronic kidney disease for dietary treatment in cardiovascular disease. J Ren Nutr.

[15] Xu H, Rossi M, Campbell KL, Sencion GL, Ärnlöv J, Cederholm T, Sjögren P, Risérus U, Lindholm B, Carrero JJ (2016). Excess protein intake relative to fiber and cardiovascular events in elderly men with chronic kidney disease. Nutr Metab Cardiovasc Dis.

[16] Chacko A, Cummings JH (1988). Nitrogen losses from the human small bowel: obligatory losses and the effect of physical form of food. Gut.

[17] de Jesús Rodríguez-Romero J, Durán-Castañeda AC, Cárdenas-Castro AP, Sánchez-Burgos JA, Zamora-Gasga VM, Sáyago-Ayerdi SG (2022). What we know about protein gut metabolites: implications and insights for human health and diseases. Food Chem.

[18] Evenepoel P, Meijers BKI, Bammens BRM, Verbeke K (2009). Uremic toxins originating from colonic microbial metabolism. Kidney Int.

[19] Meijers BK, Evenepoel P (2011). The gut-kidney axis: Indoxyl sulfate, P-cresyl sulfate and CKD progression. Nephrol Dial Transplant.

[20] Sinha AK, Laursen MF, Brinck JE, Rybtke ML, Pedersen M, Roager HM, Licht TR (2023) Substrate availability and dietary fibre regulate metabolism of tryptophan by human gut microbes. bioRxiv 2023. 10.1101/2023.06.05.543658

[21] Niwa T (2010). Indoxyl sulfate is a nephro-vascular toxin. J Ren Nutr.

[22] Rossi M, Johnson DW, Xu H, Carrero JJ, Pascoe E, French C, Campbell KL (2015). Dietary protein-fiber ratio associates with circulating levels of indoxyl sulfate and P-cresyl sulfate in chronic kidney disease patients. Nutr Metab Cardiovasc Dis.

[23] Lancaster SM, Lee-McMullen B, Abbott CW, Quijada JV, Hornburg D, Park H, Perelman D, Peterson DJ, Tang M, Robinson A, Ahadi S, Contrepois K, Hung C-J, Ashland M, McLaughlin T, Boonyanit A, Horning A, Sonnenburg JL, Snyder MP (2022). Global, distinctive, and personal changes in molecular and microbial profiles by specific fibers in humans. Cell Host Microbe.

[24] Tagliamonte S, Barone Lumaga R, De Filippis F, Valentino V, Ferracane R, Guerville M, Gandolfi I, Barbara G, Ercolini D, Vitaglione P (2023). Milk protein digestion and the gut microbiome influence gastrointestinal discomfort after cow milk consumption in healthy subjects. Food Res Int.

[25] van Eijnatten EJ, Camps G, Guerville M, Fogliano V, Hettinga K, Smeets PA (2023). Milk coagulation and gastric emptying in women experiencing gastrointestinal symptoms after ingestion of cow's milk. Neurogastroenterol Motility.

[26] Marventano S, Mistretta A, Platania A, Galvano F, Grosso G (2016). Reliability and relative validity of a food frequency questionnaire for Italian adults living in Sicily, Southern Italy. Int J Food Sci Nutr.

[27] Feunekes GI, Van Staveren WA, De Vries JH, Burema J, Hautvast JG (1993). Relative and biomarker-based validity of a food-frequency questionnaire estimating intake of fats and cholesterol. Am J Clin Nutr.

[28] Siebelink E, Geelen A, de Vries JH (2011). Self-reported energy intake by FFQ compared with actual energy intake to maintain body weight in 516 adults. Br J Nutr.

[29] Schmieder R, Edwards R (2011). Quality Control and preprocessing of metagenomic datasets. Bioinformatics.

[30] Beghini F, Mclver LJ, Blanco-Míguez A, Dubois L, Asnicar F, Maharjan S, Mailyan A, Thomas AM, Manghi P, Valles-Colomer M, Weingart G, Zhang Y, Zolfo M, Huttenhower C, Franzosa EA, Segata N (2020). Integrating taxonomic, functional, and strain-level profiling of diverse microbial communities with bioBakery 3. Elife.

[31] Buchfink B, Xie C, Huson DH (2014). Fast and sensitive protein alignment using diamond. Nat Methods.

[32] Mortazavi A, Williams BA, McCue K, Schaeffer L, Wold B (2008). Mapping and quantifying mammalian transcriptomes by RNA-seq. Nat Methods.

[33] Le Chatelier E, Nielsen T, Qin J, Prifti E, Hildebrand F, Falony G, Almeida M, Arumugam M, Batto J-M, Kennedy S, Leonard P, Li J, Burgdorf K, Grarup N, Jørgensen T, Brandslund I, Nielsen HB, Juncker AS, Bertalan M, Levenez F, Pons N, Rasmussen S, Sunagawa S, Tap J, Tims S, Zoetendal EG, Brunak S, Clément K, Doré J, Kleerebezem M, Kristiansen K, Renault P, Sicheritz-Ponten T, de Vos WM, Zucker J-D, Raes J, Hansen T, Guedon E, Delorme C, Layec S, Khaci G, van de Guchte M, Vandemeulebrouck G, Jamet A, Dervyn R, Sanchez N, Maguin E, Haimet F, Winogradski Y, Cultrone A, Leclerc M, Juste C, Blottière H, Pelletier E, LePaslier D, Artiguenave F, Bruls T, Weissenbach J, Turner K, Parkhill J, Antolin M, Manichanh C, Casellas F, Boruel N, Varela E, Torrejon A, Guarner F, Denariaz G, Derrien M, van Hylckama Vlieg JE, Veiga P, Oozeer R, Knol J, Rescigno M, Brechot C, M’Rini C, Mérieux A, Yamada T, Bork P, Wang J, Ehrlich SD, Pedersen O (2013). Richness of human gut microbiome correlates with metabolic markers. Nature.

[34] Segata N, Izard J, Waldron L, Gevers D, Miropolsky L, Garrett WS, Huttenhower C (2011). Metagenomic biomarker discovery and explanation. Genome Biol.

[35] Wickham H (2009) Introduction. ggplot2 1–7. 10.1007/978-0-387-98141-3_1

[36] R Core Team (2023) R: A language and environment for statistical computing. R Foundation for Statistical Computing, Vienna, Austria. URL https://www.R-project.org/

[37] Kuhn M (2008). Building predictive models in R using the caret package. J Stat Softw.

[38] Bolte LA, Vich Vila A, Imhann F, Collij V, Gacesa R, Peters V, Wijmenga C, Kurilshikov A, Campmans-Kuijpers MJ, Fu J, Dijkstra G, Zhernakova A, Weersma RK (2021). Long-term dietary patterns are associated with pro-inflammatory and anti-inflammatory features of the gut microbiome. Gut.

[39] Matijašić BB, Obermajer T, Lipoglavšek L, Grabnar I, Avguštin G, Rogelj I (2013). Association of dietary type with fecal microbiota in vegetarians and omnivores in Slovenia. Eur J Nutr.

[40] Tomova A, Bukovsky I, Rembert E, Yonas W, Alwarith J, Barnard ND, Kahleova H (2019). The effects of vegetarian and vegan diets on gut microbiota. Front Nutr.

[41] De Filippis F, Pellegrini N, Vannini L, Jeffery IB, La Storia A, Laghi L, Serrazanetti DI, Di Cagno R, Ferrocino I, Lazzi C, Turroni S, Cocolin L, Brigidi P, Neviani E, Gobbetti M, O’Toole PW, Ercolini D (2015). High-level adherence to a Mediterranean diet beneficially impacts the gut microbiota and associated metabolome. Gut.

[42] Amaretti A, Gozzoli C, Simone M, Raimondi S, Righini L, Pérez-Brocal V, García-López R, Moya A, Rossi M (2019). Profiling of protein Degraders in cultures of human gut microbiota. Front Microbiol.

[43] Carroll IM, Ringel-Kulka T, Ferrier L, Wu MC, Siddle JP, Bueno L, Ringel Y (2013). Fecal protease activity is associated with compositional alterations in the intestinal microbiota. PLoS ONE.

[44] Barrios C, Beaumont M, Pallister T, Villar J, Goodrich JK, Clark A, Pascual J, Ley RE, Spector TD, Bell JT, Menni C (2015). Gut-microbiota-metabolite axis in early renal function decline. PLoS ONE.

[45] Hall AB, Yassour M, Sauk J, Garner A, Jiang X, Arthur T, Lagoudas GK, Vatanen T, Fornelos N, Wilson R, Bertha M, Cohen M, Garber J, Khalili H, Gevers D, Ananthakrishnan AN, Kugathasan S, Lander ES, Blainey P, Vlamakis H, Xavier RJ, Huttenhower C (2017). A novel ruminococcus gravus clade enriched in inflammatory bowel disease patients. Genome Med.

[46] Turnbaugh PJ, Ley RE, Mahowald MA, Magrini V, Mardis ER, Gordon JI (2006). An obesity-associated gut microbiome with increased capacity for Energy Harvest. Nature.

[47] Ley RE, Bäckhed F, Turnbaugh P, Lozupone CA, Knight RD, Gordon JI (2005). Obesity alters gut microbial ecology. Proc Natl Acad Sci.

[48] Garcia-Mantrana I, Selma-Royo M, Alcantara C, Collado MC (2018). Shifts on gut microbiota associated to Mediterranean diet adherence and specific dietary intakes on general adult population. Front Microbiol.

[49] Dietrich S, Trefflich I, Ueland PM, Menzel J, Penczynski KJ, Abraham K, Weikert C (2022). Amino acid intake and plasma concentrations and their interplay with gut microbiota in vegans and omnivores in Germany. Eur J Nutr.

[50] De Filippis F, Pasolli E, Ercolini D (2020). The food-gut axis: lactic acid bacteria and their link to food, the gut microbiome and human health. FEMS Microbiol Rev.

[51] Kaoutari AE, Armougom F, Gordon JI, Raoult D, Henrissat B (2013). The abundance and variety of carbohydrate-active enzymes in the human gut microbiota. Nat Rev Microbiol.

[52] Chung WS, Walker AW, Vermeiren J, Sheridan PO, Bosscher D, Garcia-Campayo V, Parkhill J, Flint HJ, Duncan SH (2018). Impact of carbohydrate substrate complexity on the diversity of the human colonic microbiota. FEMS Microbiol Ecol.

[53] Bohra V, Dafale NA, Purohit HJ (2019). Understanding the alteration in rumen microbiome and CAZymes profile with diet and host through comparative metagenomic approach. Arch Microbiol.

[54] Ye S, Shah BR, Li J, Liang H, Zhan F, Geng F, Li B (2022). A critical review on interplay between dietary fibers and gut microbiota. Trends Food Sci Technol.

[55] Wardman JF, Bains RK, Rahfeld P, Withers SG (2022). Carbohydrate-active enzymes (CAZymes) in the gut microbiome. Nat Rev Microbiol.

[56] Haro C, Rangel-Zúñiga OA, Alcalá-Díaz JF, Gómez-Delgado F, Pérez-Martínez P, Delgado-Lista J, Quintana-Navarro GM, Landa BB, Navas-Cortés JA, Tena-Sempere M, Clemente JC, López-Miranda J, Pérez-Jiménez F, Camargo A (2016). Intestinal microbiota is influenced by gender and body mass index. PLoS ONE.

[57] Takagi T, Naito Y, Inoue R, Kashiwagi S, Uchiyama K, Mizushima K, Tsuchiya S, Dohi O, Yoshida N, Kamada K, Ishikawa T, Handa O, Konishi H, Okuda K, Tsujimoto Y, Ohnogi H, Itoh Y (2018). Differences in gut microbiota associated with age, sex, and stool consistency in healthy Japanese subjects. J Gastroenterol.

