# Supplementary material for: Relationships between diet and gut microbiome in an Italian and Dutch cohort: does the dietary protein to fiber ratio play a role?
Source: Eur J Nutr. 2023 Dec 27;63(3):741–50. doi: 10.1007/s00394-023-03308-4 (PMC10948488; doi:10.1007/s00394-023-03308-4)
Supplement: Supplementary file 1 — Supplementary file1 (DOCX 590 KB) [file 394_2023_3308_MOESM1_ESM.docx]

**Relationships between diet and gut microbiome in an Italian and Dutch cohort: does the dietary protein to fiber ratio play a role?**

Silvia Tagliamonte^1^, Marie-Luise Puhlmann^3,4^, Francesca De Filippis^1,2^, Mathilde Guerville^5^, Danilo Ercolini^1,2^, Paola Vitaglione^1,2^

^1^ Department of Agricultural Sciences, University of Naples Federico II, 80055 Portici, Italy

^2^ Task Force on Microbiome Studies, University of Naples Federico II, 80134 Naples, Italy

^3^ Division of Human Nutrition and Health, Wageningen University & Research, Wageningen, The Netherlands

^4^ Laboratory of Microbiology, Wageningen University & Research, Wageningen, The Netherlands

^5^ Nutrition Department, Lactalis Research & Development, 35240 Retiers, France

***Corresponding author:**

Prof Paola Vitaglione, PhD

Department of Agricultural Sciences, University of Naples “Federico II”

Parco Gussone Ed. 84, Portici (NA), 80055, Italy

Telephone: +39 081 2539357;

E-mail: [*paola.vitaglione@unina.it*](mailto:paola.vitaglione@unina.it)

**Supplementary Table 1:** Habitual energy intake from macronutrients of Italian female cohort (F_IT), Dutch female cohort (F_NL), Italian male cohort (M_IT).

|  | **Italian females**  **(n=20)** | **Dutch females (n=30)** | **Italian males**  **(n= 19)** |
| --- | --- | --- | --- |
| % Energy from proteins (%) | 18.8 ± 0.54^a^ | 14.0 ± 0.35^b^ | 17.7 ± 0.58^a^ |
| % Energy from fats (%) | 37.0 ± 1.87^a^ | 37.3 ± 0.87^a^ | 36.6 ± 1.10^a^ |
| % Energy from carbohydrates (%) | 40.1 ± 1.76^a^ | 42.6 ± 0.85^a^ | 42.6 ± 1.42^a^ |
| % Energy from fibers (%) | 3.0 ± 0.21^a^ | 2.5 ± 0.07^b^ | 1.9 ± 0.15^c^ |

Different letters indicate p<0.05 assessed by One-way ANOVA and Tukey’s *post hoc* test. Data are expressed as means ± SEM.


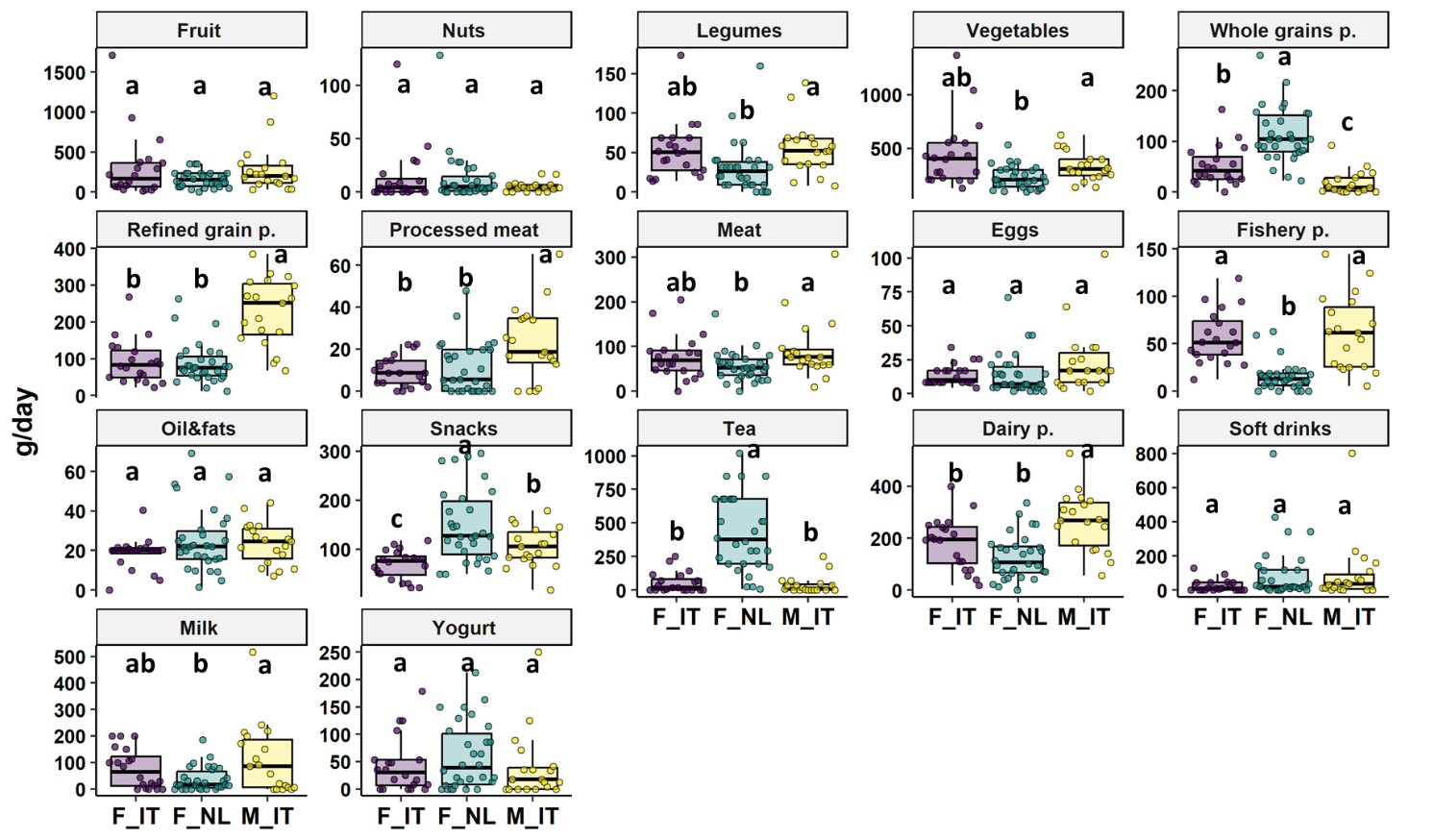


**Supplementary Fig. 1:** Habitual food categories intake in Italian female cohort (F_IT; light violet), Dutch female cohort (F_NL; teal), Italian male cohort (M_IT; yellow). Different letters indicate differences between samples (One-way ANOVA and Tukey’s *post hoc* or Mann–Whitney, *p* < 0.05 depending on data normal distribution).


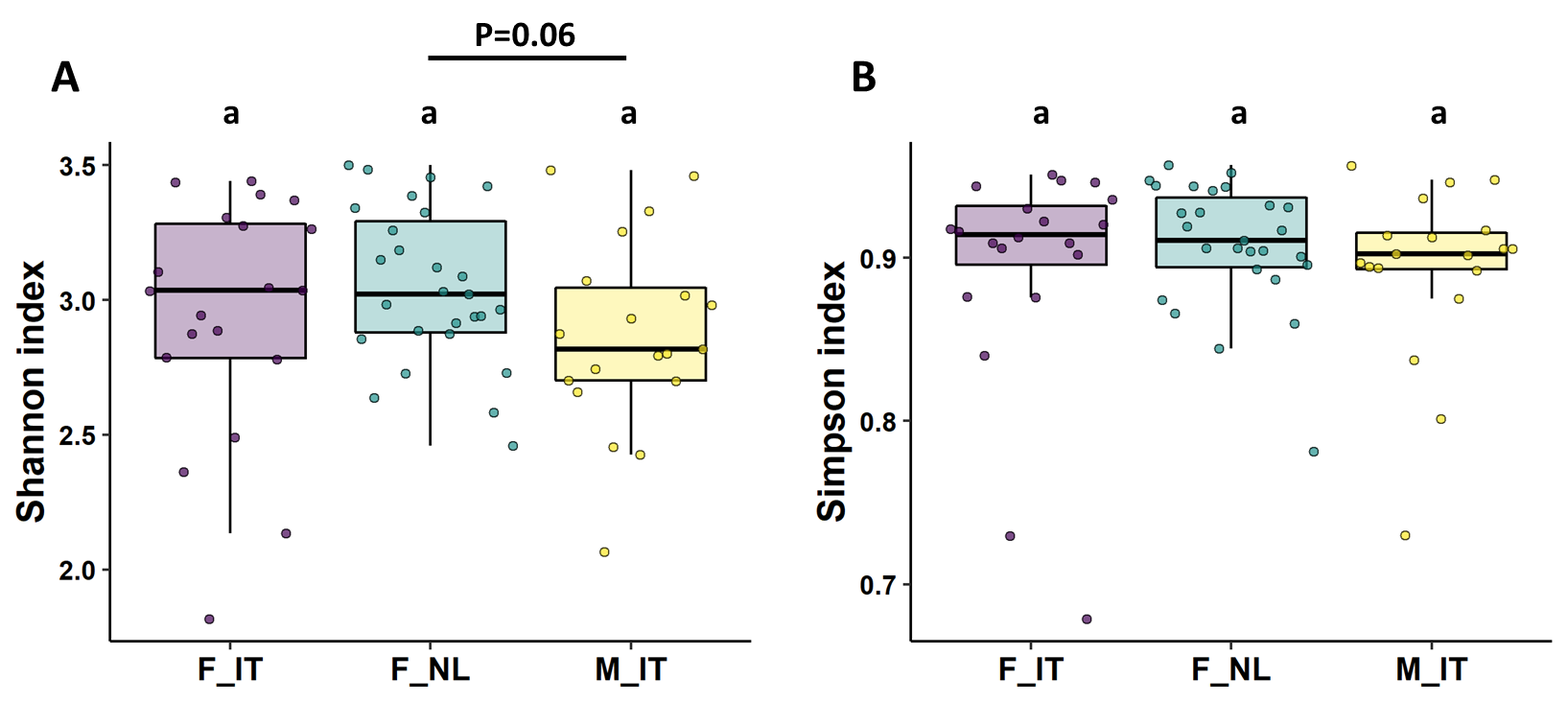


**Supplementary Fig. 2:** Microbial shannon and simpson index in Italian female cohort (F_IT; light violet), Dutch female cohort (F_NL; teal), Italian male cohort (M_IT; yellow). Different letters indicate differences between samples (Wilcoxon test, *p* < 0.05).
